# Nitric Oxide-Releasing Platforms for Treating Cardiovascular Disease

**DOI:** 10.3390/pharmaceutics14071345

**Published:** 2022-06-25

**Authors:** Mingyue He, Deping Wang, Yumei Xu, Fangying Jiang, Jian Zheng, Yanlin Feng, Jimin Cao, Xin Zhou

**Affiliations:** 1 Department of Medical Imaging, Shanxi Medical University, Taiyuan 030001, China; hemy1005@163.com (M.H.); xym_jsnt@163.com (Y.X.); 2 Key Laboratory of Cellular Physiology, Ministry of Education, The Department of Physiology, Shanxi Medical University, Taiyuan 030001, China; wangdeping@sxmu.edu.cn (D.W.); jfy54264@163.com (F.J.); zhengjiansxmu@126.com (J.Z.); 3 Department of Breast Surgery, Shanxi Provincial Cancer Hospital, Shanxi Medical University, Taiyuan 030001, China

**Keywords:** nitric oxide, controlled release, cardiovascular disease, biomaterials, synergetic therapy

## Abstract

Cardiovascular disease (CVD) is the first leading cause of death globally. Nitric oxide (NO) is an important signaling molecule that mediates diverse processes in the cardiovascular system, thereby providing a fundamental basis for NO-based therapy of CVD. At present, numerous prodrugs have been developed to release NO in vivo. However, the clinical application of these prodrugs still faces many problems, including the low payloads, burst release, and non-controlled delivery. To address these, various biomaterial-based platforms have been developed as the carriers to deliver NO to the targeted tissues in a controlled and sustained manner. This review aims to summarize recent developments of various therapeutic platforms, engineered to release NO for the treatment of CVD. In addition, two potential strategies to improve the effectiveness of existing NO therapy are also discussed, including the combination of NO-releasing platforms and either hydrogen sulfide-based therapy or stem cell therapy. Hopefully, some NO-releasing platforms may provide important therapeutic benefits for CVD.

## 1. Introduction

Cardiovascular disease (CVD) is the most common disease, endangering the health of people and causing about 19 million deaths globally in 2020, and thus has become the first killer of human life and health in the world [1]. Patients with CVD sometimes need not only long-term medication (such as hypertension) but also some high-risk operations (such as cardiomyopathy and myocardial infarction) in severe cases. According to the report of the American Heart Association in 2022, the direct and indirect costs of CVD in the United States from 2017 to 2018 are about USD 378.0 billion, accounting for 12% of the total medical expenses in the United States [1]. Therefore, CVD is not only a huge medical burden but also a heavy economic and social burden. How to develop an effective and minimally invasive treatment under the current medical conditions is an urgent task for researchers.

Nitric oxide (NO) is a small-molecule gas, which has paracrine and autocrine functions and serves as an important regulatory factor of the circulatory system [2]. It was first found as an endothelium-derived relaxing factor (EDRF), which plays a key role in vasodilation [3]. Based on this important discovery of NO, Robert Furchgott, Louis Ignarro, and Ferid Murad shared the Nobel Prize in medicine in 1998 [4]. Besides affecting vascular tension, NO also has the functions of anti-inflammatory, anti-oxidation, reducing blood lipid levels, inhibiting vascular smooth muscle cells (SMCs) proliferation, and platelet aggregation [5]. Endogenous NO can be generated by catalyzing L-arginine with nitric oxide synthetase (NOS), including endothelial nitric oxide synthetase (eNOS), neuronal nitric oxide synthetase (nNOS), and inducible nitric oxide synthetase (iNOS) [6]. For the cardiovascular system, eNOS is a vital regulator of cardiovascular homeostasis, because it is the main source of NO synthesis in vascular endothelium [7]. In a physiological state, NO produced by eNOS can inhibit the expression of adhesion factors and platelet aggregation to reduce the risk of early atherosclerosis [8]. However, some pathological conditions, such as continuous cardiac pressure overload, can induce eNOS uncoupling, resulting in a decrease in NO production and finally aggravating the disease [9]. Therefore, the addition of exogenous NO can be beneficial to prevent and cure CVD.

How to deliver exogenous NO in vivo is still a challenge, due to the difficulties in handling and transporting NO gas. For example, NO is a gas molecule with a short half-life and instability, which makes its influence range only a few microns, limiting the effectiveness of NO reaching the target site [10]. Moreover, NO can react with several intracellular targets indiscriminately, excessive reactive oxygen species (ROS) to generate peroxynitrite, which further leads to nitrite stress and even tissue damage [11,12,13]. In addition, the physiological effects of NO are concentration dependent, with a proangiogenic effect at low concentrations (10^−12^–10^−9^ M) and a proapoptotic effect at high concentrations (10^−6^ M) [14]. To solve the problems described above and improve the treatment efficacy of NO, much effort has been focused on developing feasible and effective means for NO delivery in a timely, targeted, quantitative, and stable manner.

The development of NO donor, which is the pharmacologically active carrier of NO, can assist in the in situ release of NO in the target tissues [15]. The release of NO from the donor molecule can be triggered by factors such as light, heat, pH change, or enzyme activity. For example, sodium nitroprusside (SNP) was the first metal–NO compound discovered 150 years ago and was first used as an anti-hypertensive drug successfully in 1970 [16]. It is still widely used in modern medicine, as a common vasodilator in cardiac surgery, hypertension emergency, heart failure, and vascular surgery [17]. NO produced by SNP can stimulate guanylate cyclase to produce cyclic guanosine monophosphate (cGMP), thus relaxing smooth muscle, increasing cardiac blood flow, and lowering blood pressure [17]. However, there are still some shortcomings in the use of NO donors. For example, although SNP is stable enough to release NO spontaneously under the influence of light, it cannot release NO continuously for a long time, resulting in the insufficient release of NO, thus affecting the curative effect of CVD [18,19]. To stabilize the NO release from donors, it is feasible to load the NO donors onto the biomaterials to prepare a NO delivery system. At present, however, only a few products of NO delivery have received approval from the United States Food and Drug Administration (FDA) [20].

Considering the increasingly important role of NO in gas therapy for CVD, this review aims to highlight recent developments of various therapeutic platforms, engineered to release NO for the treatment of CVD, including NO drugs and NO-releasing biomaterials. In addition, two potential strategies to improve the effectiveness of existing NO therapy are also discussed, including the combination of NO-releasing platforms and either hydrogen sulfide (H_2_S)-based therapy or stem cell therapy. Recently, there have been several reviews on the delivery of NO for treating CVD [14,20,21]. Compared to these review works, this is the first comprehensive review on different levels of NO-releasing platforms, from NO donors or NO-related drugs to NO-releasing biomaterials to NO-based synergistic therapy for the treatment of CVD. Moreover, this review also introduces the most recent research achievements in this area. Hopefully, some NO-releasing platforms may provide important therapeutic benefits for CVD.

## 2. The Mechanism of NO in CVD and NO-Related Drugs

**Figure 1:**
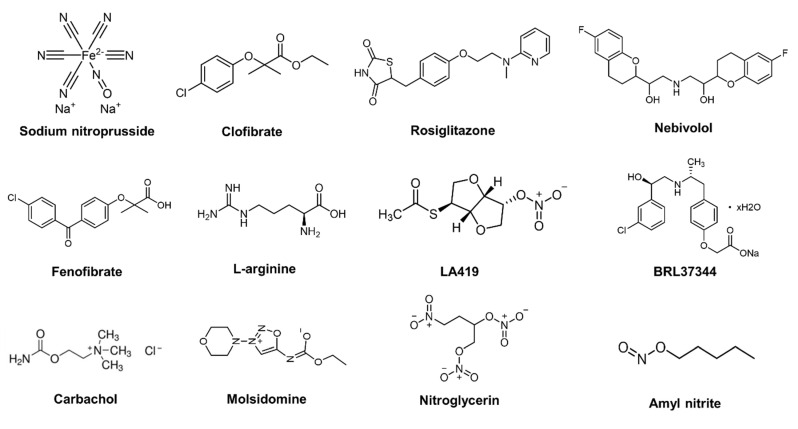
Chemical structures of selected NO donors or NO-related drugs.

Adults can inhale NO gas for a variety of therapeutic purposes. However, NO gas therapy efficacy was affected by the characteristics of NO, such as extremely short half-lives, aimless distribution, and difficulty accumulating in target tissues. To address this issue, several NO donors or NO-related drugs that directly release NO or indirectly increase the concentration of NO have been developed. There are four major types of low-molecular-weight NO donors that can produce NO exogenously: N-diazeniumdiolates (NONOates), S-nitrosothiols (RSNO), metal–nitrosyl complexes, and nitrobenzene. Among them, the most extensively studied NO donor is NONOate, which spontaneously decomposes under physiological conditions, releasing two moles of NO for every mole of donor [22]. In comparison, RSNO is an endogenous NO donor and functions as a physiological transporter of NO in vivo, such as S-nitroso-L-cysteine (CysNO) and S-nitrosoglutathione (GSNO) [23]. RSNO releases NO under a variety of conditions, including the thermal environment, ultraviolet (UV) light, and metal ions [24,25]. For more than a decade, researchers have studied the light-induced release of NO donors such as metal–NO complexes [26] and nitrobenzene derivatives [27]. In addition to NO-releasing donor molecules, there are also some NO-related drugs which can improve NO production indirectly; for instance, by increasing the expression of eNOS or activating eNOS. These NO donors or NO-related drugs have been widely used in the treatment of CVD, including hypertension, myocardial infarction, left ventricular (LV) hypertrophy, and heart failure (Table 1). The chemical structures of these compounds are shown in Figure 1.

### 2.1. Hypertension

Hypertension is a common cardiovascular disease and a major risk factor for cardiovascular and cerebrovascular diseases. Some patients even need to take medication for a lifetime to control their blood pressure. Hypertension patients usually have the characteristics of increased vascular tension, vascular remodeling, and vascular dysfunction caused by endothelial cell damage and increased oxidative stress [51,52,53]. The decrease in NOS/NO/cGMP cascade activity will lead to hypertension [7]. In patients with hypertension or prehypertension, the effect of endothelium-dependent vasodilators, such as acetylcholine, is weakened, while SNP is a direct NO donor independent of the endothelium, which releases NO through endothelial cell metabolism. SNP can reduce blood pressure rapidly and thus is often used to treat hypertension [28]. Patients treated with SNP can still achieve a good vasodilation effect [7,54]. Although SNP has a rapid anti-hypertensive effect, it cannot be used in the treatment of chronic hypertension due to tolerance problems, systemic hypotension, and reflex tachycardia [55].

In recent years, some studies have shown that peroxisome proliferator-activated receptors (PPARs) play an important role in the regulation of endothelial function. There are two forms of blood pressure reduction by PPARs: PPARα and PPARγ. PPARα agonists can improve the expression of eNOS, and then increase the content of NO. They also can decrease the content of endothelin-1 (ET-1) and inhibit the activity of nicotinamide adenine dinucleotide phosphate (NADPH) oxidase, thus reducing blood pressure. PPARγ agonists can downregulate the angiotensin (ang) II receptor, reduce the content of ET-1, increase the production of NO, and achieve the effect of lowering blood pressure [56]. For example, clofibrate is an important PPARα activator that stimulates the expression and activity of eNOS by activating PPARα in endothelial cells (ECs), thus increasing the production of NO [43]. It has been proved that clofibrate can significantly increase eNOS protein expression and enzyme activity in the left ventricle and improve endothelium-dependent vasodilation in hypertensive rats [29]. PPARγ agonists (rosiglitazone for instance) can upregulate nuclear factor erythroid-2-related factor 2 (Nrf2) in the positive feedback loop to maintain transcription factor expression, thus improving the bioavailability of NO in ECs [36]. The activation of eNOS and increased NO production were observed in young male spontaneously hypertensive rats (SHR) when treated with rosiglitazone [43].

### 2.2. Myocardial Infarction

Acute myocardial infarction refers to the myocardial necrosis caused by acute and persistent occlusion of the coronary artery. It can be complicated with arrhythmia, shock, or heart failure, and can even be life threatening. The main cause of myocardial infarction is the rupture of unstable atherosclerotic plaque, which leads to the aggregation of platelets on the surface of the plaque, followed by the formation of thrombus and the obstruction of the lumen of the larger coronary artery, ultimately resulting in myocardial ischemia and necrosis. Treatments with exogenous NO donors benefit patients with atherosclerosis and myocardial infarction. Nitroglycerin, a classical NO donor drug, has been clinically applied for more than 100 years and is still a standard vasodilator [30]. It was recently suggested that intermittent treatment with the short-acting nitroglycerin could significantly decrease the infarct size by promoting coronary collateral development [31]. Amyl nitrite is another classical NO donor drug and was first used to treat coronary heart disease in 1867. Its pharmacological effects are similar to nitroglycerin, lowering blood pressure and treating angina pectoris [32]. Moreover, molsidomine, a NO-donating drug, can spontaneously release NO and decrease the plasma level of soluble adhesion molecule ICAM-1, which may slow down the progression of atherosclerosis [57]. A recent study showed that molsidomine can improve heart function, enhance the stability of atherosclerotic plaque, and reduce the occurrence of myocardial infarction [34].

β-adrenergic receptors (β-ARs) play a key role in regulating cardiovascular activity [58,59,60]. In some cases, β-ARs can directly affect the production of NO. Nebivolol, a highly selective third-generation β-receptor blocker and also the β-adrenergic antagonist, causes vasodilatation primarily by activating eNOS, thus having a NO-mediated effect [37]. A study has shown that nebivolol can treat LV dysfunction after myocardial infarction in mice [38]. The main mechanism of treatment of early LV dysfunction after myocardial infarction is that nebivolol can prevent NADPH oxidase activation and enhance eNOS-dependent NO utilization [38]. Nebivolol also has antioxidant properties, which can prevent the uncoupling of NOS, thus improving the bioavailability of NO [39,40,41]. Fenofibrate, a PPARα agonist, has been found to increase the expression of eNOS in ECs [44]. Fenofibrate may prevent endothelial dysfunction by reducing ROS production and up-regulating tetrahydrobiopterin, which is an essential cofactor of NOS [43]. Fenofibrate may also have the effect of reconnecting eNOS to protect against atherosclerosis and endothelial dysfunction, and finally preventing myocardial infarction [45]. Furthermore, LA419, a NO donor, has been proved to have anti-ischemia, anti-thrombosis, and anti-atherosclerosis effects, so it can be used in the treatment of myocardial infarction [46].

### 2.3. Left Ventricular Hypertrophy

Patients with hypertension for a long time may suffer from left ventricular hypertrophy (LVH), which is also a complication of hypertension [61,62,63]. The pathogenesis of LVH is multifactorial. On the one hand, the main pathogenesis of hypertensive LVH can be related to hemodynamic factors, which are correlated with the increase in LV afterload. Compensatory hypertrophy of cardiomyocytes makes the ventricular wall gradually thicker in order to overcome resistance and adapt to pressure. On the other hand, some non-hemodynamic factors, such as the renin–angiotensin system, catecholamine, sexual factors, and genetic and metabolic factors, can also participate in the pathogenesis of LVH [64,65]. It has been suggested that NO has a certain resistance to the procession of LVH caused by essential hypertension [66]. L-arginine is a precursor of NO production. When O_2_, NADPH, FAD, FMN, and BH4 are present, eNOS forms a complex with Ca^2+^-calmodulin to catalyze the conversion of L-arginine to L-citrulline and release NO, which can reverse several markers in LVH [47,48]. A previous study has confirmed that NO has an effect of anti-cardiac hypertrophy in spontaneously hypertensive rats (SHR) after long-term administration of L-arginine [49]. In recent years, it has been demonstrated that L-arginine administration can enhance the expression of eNOS in the myocardium, resulting in the amelioration of oxidative and hemodynamic parameters in rats with LVH [67]. NO can also prevent LVH progression by reducing blood pressure and interacting with the renin–angiotensin–aldosterone system, sympathetic nervous system, and other systems [68]. LA419, a NO donor, plays a beneficial role in preventing the progression of inadaptable cardiac hypertrophy [28]. In addition, it has been reported that β_3_-AR agonists can treat LVH. For example, BRL37344 (a β_3_-AR agonist) can improve LV systolic and diastolic dysfunction and has been used in the treatment of cardiac hypertrophy, due to the increase in nNOS protein expression and NO production, as well as the inhibition of superoxide anion generation [37].

### 2.4. Heart Failure

The final stage of heart disease development is heart failure. Without treatment or intervention, almost all cases of heart diseases can develop into heart failure. Loss of NO activity in peripheral and coronary vessels is an important cause of heart failure [7]. Research shows that the bioavailability of NO in the coronary arteries of failing hearts is decreased [69]. NO regulation can be enhanced by activating the eNOS/nNOS pathway and reducing oxidative stress, consequently preventing heart failure [69]. For example, carbachol can mediate the production of NO from nNOS and thus protect the heart [51]. The main causes of chronic heart failure (CHF) are coronary heart disease, hypertension, and dilated cardiomyopathy. Activation of β_3_-AR in a NO-dependent manner has a cardioprotective effect [51,70]. For example, the aforementioned drug nebivolol can treat myocardial infarction and improve LV function in CHF [42].

**Figure 2:**
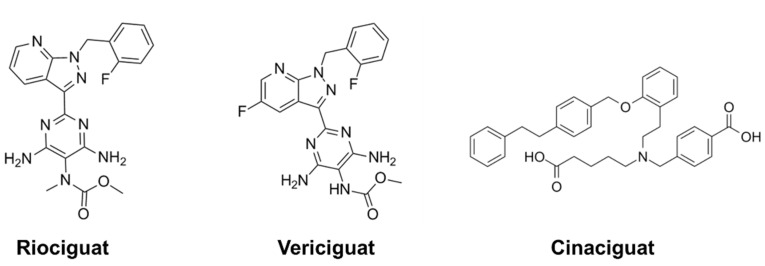
Chemical structures of selected sGC stimulators.

Although NO donors or NO-related drugs have been widely used in CVD treatments, there are several drawbacks, such as short effective reaction time and poor specificity. With the deepening of research related to the mechanisms of the NO-soluble guanylate cyclase(sGC)-cGMP signaling pathway, the clinical application of sGC stimulators in the cardiovascular system has been widely carried out. The cardiovascular protective effects of sGC stimulators are independent of NO concentration, and sGC stimulators can activate sGC directly to catalyze the generation of cGMP, which can regulate the physiological processes in the cardiovascular system to achieve therapeutic purposes [71]. Recently, a number of sGC stimulators have been developed and their roles in the cardiovascular system have been investigated (Figure 2). For example, riociguat, a classic sGC stimulator, is approved for the treatment of pulmonary hypertension and chronic thromboembolic pulmonary hypertension by inducing pulmonary vasorelaxation which results in an increase in pulmonary blood flow and a decrease in pulmonary blood pressure [72,73]. Vericiguat, a novel sGC stimulator, has been shown to directly stimulate sGC and enhance sGC sensitivity to endogenous NO in a randomized, double-blind, controlled clinical trial [74]. Vericiguat can be used to treat heart failure with reduced ejection fraction [75]. In clinical trials, Lombardi et al. demonstrated the superior efficacy of vericiguat in patients at high risk of heart failure, reducing the incidence of cardiovascular death or hospitalization for heart failure [76]. Recently, Veres et al. found that cinaciguat, an sGC stimulator, has a protective effect on cardiac ischemia-reperfusion injury in patients with graft vessels. Cinaciguat can enhance cGMP signaling and significantly reduce DNA breakage and nitrogen oxidative stress, and thus may benefit vascular endothelial dysfunction in patients with arterial bypass graft during cardiac surgery [77].

In addition to the direct and indirect release of NO by various drugs, some in vivo and in vitro heart treatment devices are also indispensable. Stents and catheters are commonly used in the treatment of CVD, which often require the participation of various materials to meet different treatment needs. Therefore, it is still a hot topic to develop different biomedical devices. The following will discuss NO-related therapeutics in the treatment of CVD using biomaterials-based strategies.

## 3. Application of NO-Releasing Biomaterial Platforms in CVD Treatment

**Figure 3:**
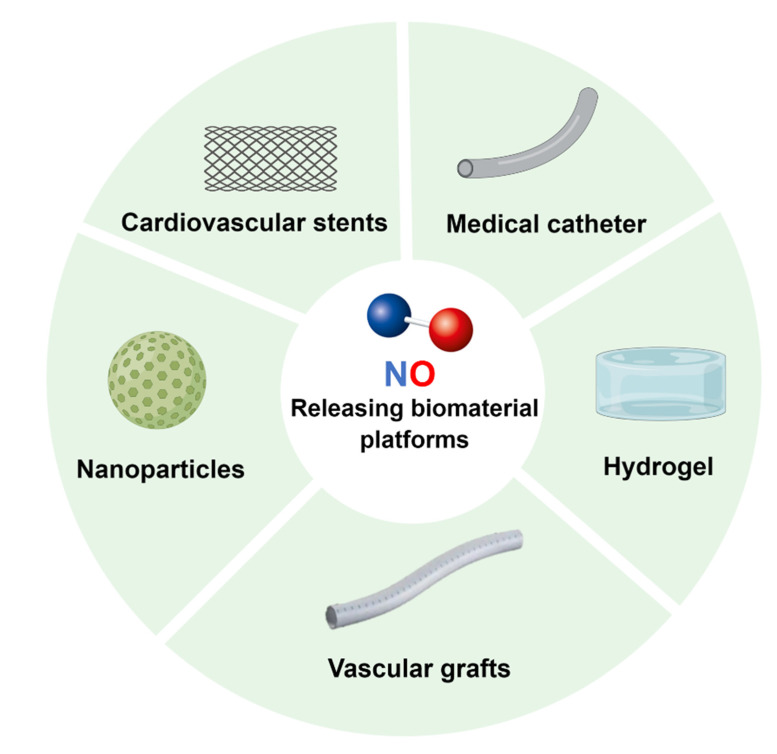
A schematic summary of the different biomaterial platforms that have been engineered to store and release NO for CVD treatments.

Although these above-mentioned NO donors have been used in clinical applications, there are many limitations that remain to be addressed, including the short half-life, instability, cytotoxicity, and limited payload of NO. Thus, researchers are devoted to developing carrier biomaterials for loading NO donors to release NO accurately and effectively [15]. At present, there are a variety of NO-releasing biomaterial platforms for the treatment of CVD, such as the surface coating of medical devices such as stents and catheters, polymer-based nanoparticles with controllable size and good stability, and flexible, hydrophilic, and adjustable hydrogels. Their applications in cardiovascular diseases are described in detail below (Figure 3).

### 3.1. Cardiovascular Stents

In the treatment of cardiovascular diseases, interventional therapy is indispensable for cardiac surgery, such as implanting cardiovascular stents in the diseased area. At present, bare-metal stents (BMSs) and drug-eluting stents (DESs) are the most commonly used in interventional therapy for cardiovascular disease, and in previous studies, it was generally believed that the outcomes of using DESs would be superior to that of BMSs [78]. Although the use of DESs can effectively reduce the incidence of in-stent restenosis, it also has some defects, such as the induced risk of late acute thrombosis by delaying re-endothelialization which reduces the long-term effectiveness of interventional therapy [79]. Therefore, how to develop safe and effective cardiovascular stents has become a research hotspot. A lot of effort has been made to accurately mimic native endothelial functions on cardiovascular stents, such as NO generation.

Metal–organic framework (MOF) is a new type of coordination material composed of a metal junction and organic bridging ligand, with the advantages of adjustable pore size, shape, structure, and biodegradation [15,80]. Excellent MOF porous solid materials such as MIP-177 not only have a higher NO adsorption capacity than other porous solid materials but also have the ability to release NO to drive controlled cellular mitochondrial respiration and stimulate cell migration [81]. MOF also has good storage stability and magnetic response function of MOF equipped with a NO carrier, with burst and steady release of two NO release stages [82]. In addition, some MOF materials, after binding with NO donors, impart MOF-based medical devices with antibacterial properties, which can be used as an efficient tool for the early prevention of medical device biofilm formation. Garren et al. developed a multifunctional three-layer composite scaffold with H_3_[(Cu_4_Cl)_3_(BTTri)_8_-(H_2_O)_12_]·72H_2_O and a NO donor, S-nitroso-N-acetylpenicillamine (SNAP). This scaffold exhibited enhanced NO release and had a good antibacterial effect, significantly reducing the adhesion of *Staphylococcus aureus* and *Escherichia coli* [83]. Copper ion (Cu^II^*)* can promote the growth of ECs and increase the content of NO in vivo. Fan et al. prepared a kind of nano Cu-MOF on the surface of titanium, which can catalyze the production of NO and the transfer of Cu^II^ at the same time. This kind of Cu-MOF coating has anti-proliferation ability against VSMCs and macrophages, thus reducing neointimal hyperplasia. At the same time, it can promote the re-endothelialization of cells and inhibit the formation of thrombus, so as to treat cardiovascular diseases [80].

Recently, the emerging bionic surface-modified cardiovascular stents have the function of improving blood compatibility and reducing the restenosis of stents in the later stages, which has become a hot topic in recent years [84]. Metal–catecholamine assembly strategy is a kind of biomimetic coating. Li et al. used 3,4-dihydroxy-l-phenylalanine (DOPA) to crosslink selenocysteine (SecA) and Cu^II^ to prepare a Cu^II^ DOPA/SecA coating, which can mimic the catalytic activity of glutathione peroxidase to realize long-term, stable, adjustable NO release. This kind of NO-releasing coating can promote re-endothelialization, suppress thrombosis, and reduce intimal hyperplasia in vivo [85]. In addition, Zhang et al. obtained a DA-Cu^II^ coating by immersing the scaffold in an aqueous solution containing dopamine (DA) and Cu^II^. The coating can catalyze the decomposition of the natural NO donor RSNO in the circulating blood, and then release NO in a local stable, long-term, and controllable way. DA-Cu^II^ coating has specific cell selectivity and can inhibit the growth and migration of VSMCs, which can also promote the re-endothelialization of cells, inhibit the formation of thrombus, and improve the anti-restenosis performance of stents in vivo [86].

**Figure 4:**
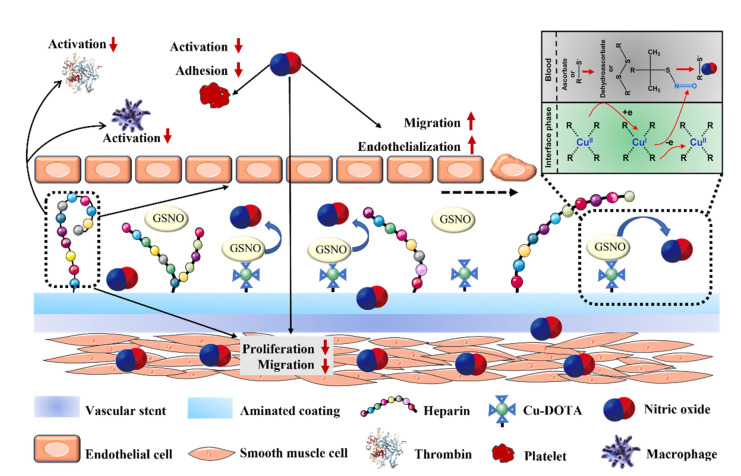
Schematic diagram of the durable endothelium-mimicking coating on stents. The coating is developed by covalently immobilizing Cu–DOTA coordination complexes (NO-producing substances) and heparin. By combining NO and heparin, the coating inhibits thrombogenic responses and the proliferation of VSMCs, thereby reducing the risk of thrombosis and stenosis of the stent. Upward arrows in red represent enhancement, and downward arrows in red represent inhibition. Reproduced with permission from [87], Bioactive Materials, 2021.

How to realize the continuous and controllable release of NO by coatings is an important research focus in the modification of vascular stents. In a recent study, the Huang and Yang lab reported a durable endothelium-mimicking coating on stents by binding a NO-producing substance Cu-DOTA (1,4,7,10-tetraazacyclododecane-N,N′,N′′,N′′′-tetraacetic acid) and bioactive molecule heparin (Figure 4). The surface of the scaffold was first coated with polydopamine (pDA), followed by surface chemical crosslinking with polyamines (pAM) to form a long-lasting mussel-inspired amine-bearing adhesive coating (pAMDA), and then covalently fixed Cu-DOTA with NO release function and heparin with anticoagulant function by a grafting method. The high chemical stability of the pAMDA coating maximizes the biological activity of heparin while continuing to release NO for up to a month. Therefore, the functionalized vascular stent exhibits a long-term physiological effect of mimicking the endothelium in inhibiting thrombosis, inflammation, and immunity, thereby effectively reducing in-stent restenosis [87].

Another method of sustained and localized on-demand release of NO, besides endogenous NO, is to incorporate NO donors into biomaterials via physical or chemical approaches [88]. Zhu et al. developed a bifunctional stent with anticoagulant and endothelial functions. A block copolymer brush with a zwitterionic structure is grafted to the bare metal coronary stent surface by an atom transfer radical polymerization surface, and the NO donor diethylenetriamine NONOate (DETA NONOate) is attached to the polymer brush by reactive epoxy groups to produce NO. The resulting bifunctional stent has good biocompatibility, anticoagulation, and particularly pro-endothelialization function, showing great potential for reducing postoperative restenosis and late stent thrombosis-related side effects [89].

### 3.2. Medical Catheter

Medical catheters are also commonly used in the clinical treatment of CVD. Acute catheters can be retained for up to 7 days in emergency treatment, while in the case of long-term nutrition or dialysis, some catheters may be placed permanently [90]. The biggest risk and common complications of long-term indwelling catheters are infection and thrombosis. The application of NO-releasing coating in catheters can solve these problems, due to the anti-platelet, inhibition of platelet adhesion, and anti-infection activities of NO. It has been proved that adding NO donor to polymer materials not only has no effect on the basic mechanical properties of the materials but also has the characteristics of no cytotoxin and anti-hemolysis [91]. For example, Brisbois et al. used poly(lactic-co-glycolic acid (PLGA) and diazeniumdiolated dibutylhexanediamine (DBHD/NONO) as additives in Elasteon-E2As polyurethane (PU) to prepare NO-releasing central venous catheters [92]. In the experiment, it was observed that NO-releasing materials can play an anticoagulant and antibacterial role in the long-term implantation and optimize the placement effect of catheters.

SNAP is one of the most frequently used NO donors for in vivo preclinical studies because it is relatively stable [93]. Researchers prepared a SNAP impregnated catheter by expanding the silicone rubber tube in a tetrahydrofuran solution containing SNAP [94]. This method can provide NO release at a physiological level for up to 18 days, so it has a broad application prospect in the manufacture of intravascular biosensors. Hopkins et al. covalently linked SNAP on poly(dimethylsiloxane) (PDMS) to realize a highly stable NO release for 125 days. SNAP-PDMS coating showed excellent blood compatibility and long-term antibacterial properties. It can significantly inhibit the bacterial adhesion of *Staphylococcus aureus* after one month of continuous exposure in the bioreactor. After more than 4 months of NO release, SNAP-PDMS coating still has a certain antibacterial effect and NO-releasing performance and reduces the risk of thrombosis [95]. This brings a new insight into the development of long-term NO-releasing materials.

The main reason for catheter-related bloodstream infections (CRBIs) is the long-term indwelling of the catheter in the body, which causes bacteria to adhere to the catheter, form biofilms, and resist the bactericidal effect of antibiotics [96]. In order to solve these problems, researchers have made many efforts to develop a medical catheter that can not only ensure long-term NO release but also inhibit bacterial adhesion and/or promote bacterial eradication. Pant et al. prepared a vascular catheter by combining SNAP with the hydrophobic polymer Elasteon-E2As. The catheter can not only release NO to reduce the adhesion of blood protein but also significantly inhibit the living bacteria attached to the polymer surface in the absence of cytotoxicity, showing excellent biocompatibility [97]. In another study, the Meyerhoff lab used a simple impregnation technology to add SNAP into a copolymer of silicone rubber (SR) and PU with stronger biocompatibility, and the resulting SNAP-impregnated SR-PU copolymer intravascular catheters exhibited high stability and long-term NO release performance [98]. The catheters can release NO in 14 days and have the ability to kill *Staphylococcus epidermidis* and *Pseudomonas aeruginosa*. In addition, the lab also proved that SNAP-doped SR-PU copolymer composites had anti-biofilm activities for *Pseudomonas mirabilis* [99]. In summary, the solvent impregnation method is a simple and efficient composite material synthesis method, which can improve the blood compatibility/antibacterial activity of the venous catheter and provide ideas for the development of the catheter and other implantable medical device materials.

**Figure 5:**
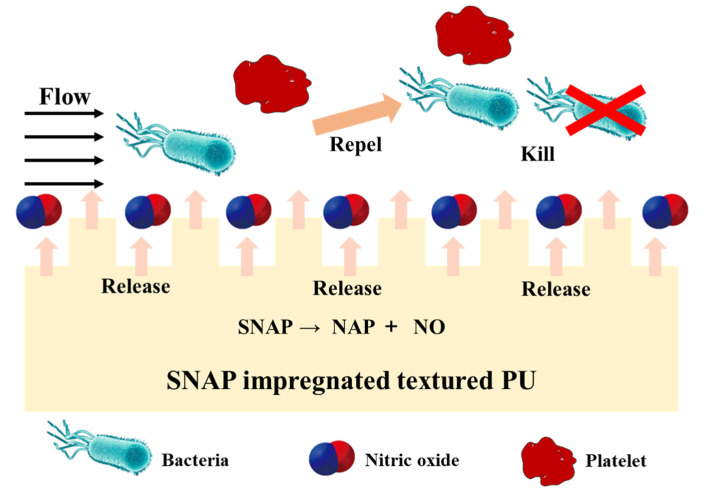
Schematic diagram of the biomimetic polyurethane (PU) biomaterials with NO-releasing property. Textured PU is impregnated with SNAP. NO releases from the surface texturing to mimic the inner surface of blood vessels, inhibiting platelets and bacterial adhesion. Reproduced with permission from [100], Acta Biomaterialia, 2019.

Because the bionic method may be able to “trick” the organism to make it no longer have a strong rejection to the foreign body entering the body and control the reaction to the foreign body surface, thus improving the biocompatibility, the bionic strategy is also crucial in the development of medical catheters. Xu et al. prepared a kind of dual-functional PU film by using a bionic strategy. This film was impregnated with SNAP as the NO donor, and its outer covering was a submicron cylindrical pattern imitating the inner surface of the blood vessel (Figure 5) [100]. Both NO release and surface texturing reduced platelet adhesion and activation, thus showing antithrombotic activities. The biomimetic surface also reduced the bacterial adhesion by the synergistic effect, thereby inhibiting biofilm formation. Because of its bionic advantages, this biomaterial may be widely used in the development of medical devices that are directly in contact with blood in the future.

### 3.3. Vascular Grafts

**Figure 6:**
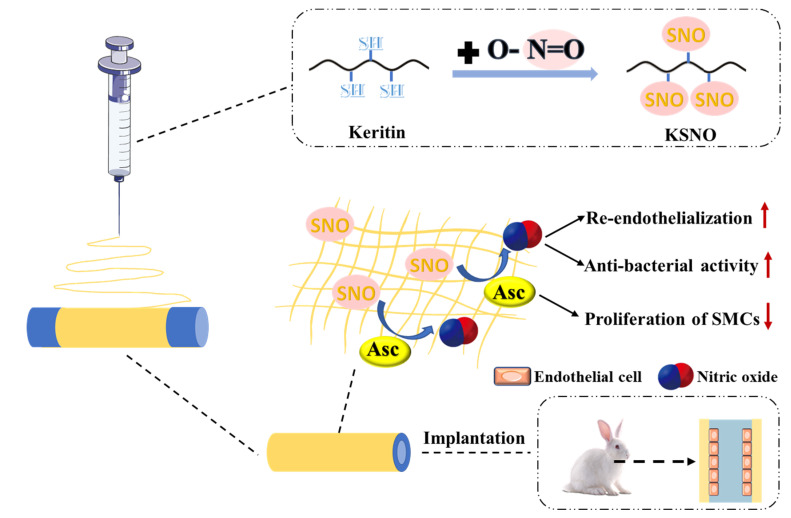
Schematic illustration of KSNO. PCL/KSNO vascular graft was prepared by co-electrospinning KSNO and PCL. NO generation, cytotoxicity, and blood compatibility, as well as the regulation of vascular cells by NO, were examined. PCL/KSNO graft was implanted and investigated in a rabbit carotid artery replacement model to evaluate the endothelialization and revascularization. Upward arrows in red represent enhancement, and downward arrows in red represent inhibition. Reproduced with permission from [101], International Journal of Biological Macromolecules, 2021.

In modern medicine, there is an urgent need for durable artificial vascular grafts with small diameters for treating vascular occlusions. In order to develop effective small-diameter artificial vessels, one of the most important steps is improving the hemocompatibility of the surface to facilitate rapid endothelialization and avoid thrombosis. In this context, using NO-releasing biomaterials would be a good strategy for the construction of vascular grafts. Li et al. synthesized a low-toxicity NO donor S-nitrosated keratin (KSNO) which could be co-electrospun with poly(ε-caprolactone) (PCL) to fabricate small-diameter vascular grafts that generate NO under the catalysis of ascorbic acid (Asc) (Figure 6) [101]. NO released from the vascular grafts can enhance the adhesion and growth of human umbilical vein endothelial cells (HUVECs). At the same time, in the presence of Asc, it can inhibit the proliferation of human aortic smooth muscle cells. Inhibition of thrombosis and complete luminal coverage by endothelial cells can be observed after 1 month of carotid artery replacement in animals. Therefore, PCL/KSNO small-diameter vascular grafts have the function of rapid endothelialization and vascular remodeling, showing potential application value in vascular tissue engineering.

Other than loading NO donors in the polymer matrix, modification of NO catalysts on the surface of vascular grafts has been confirmed to be an effective strategy for NO release in situ via catalyzing endogenous NO donors in the bloodstream [102]. For instance, organoselenium-immobilized polyethyleneimine (SePEI) has been used to modify grafts to catalyze endogenous RSNO for NO release, which can improve endothelialization and remodeling of vascular grafts [103]. Zhao’s research team reported an enzyme-mediated approach to realize the in situ NO release. Galactosidase was modified on the surface of vascular grafts to catalyze the glycosylated NO prodrug, which was intravenously administrated. The immobilized enzyme can retain biological activity after at least 1 mouth to gain stable NO release at the implantation site, which can effectively inhibit platelet adhesion and thrombus formation, whilst also improving endothelialization and vascular tissue regeneration [104]. Despite good efficacy in the in vivo experiments, this enzyme-activated approach still faces issues related to the non-specific NO release caused by the widely distributed endogenous glycosidase. In an effort to address this issue, the research team used a bump-and-hole strategy to develop a novel NO delivery system based on galactosidase-galactosyl-NONOate as an enzyme–prodrug pair, which improved the targeting of NO delivery [105]. In the hind limb ischemia and ischemia/reperfusion kidney injury (AKI) models, NO-targeted delivery exhibited improved therapeutic efficacy in tissue repair and function recovery, while side effects related to the systemic release of NO were eliminated. It may also be possible to apply this NO delivery system based on the enzyme–prodrug strategy to vascular stents and vascular grafts.

### 3.4. Nanoparticles

To avoid burst release and non-targeted delivery of NO, one effective strategy is to encapsulate NO donors into nanoparticles (NPs). The application of nanomaterials to deliver NO has the following advantages: Firstly, the nanomaterials can stably load a large amount of NO with high bioactivity; secondly, the surface of nanoparticles can be chemically modified and optimized to meet different medical needs; thirdly, encapsulation of NO donors in nanocarriers enable controlled and targeted delivery of NO [106,107]. Zhang et al. developed a type of NO-releasing gelatin-siloxane nanoparticle (GS NP) via modifying RSNO on GS NPs [108]. The synthesized GS-NO NPs not only released NO continuously for 7 days but also had good water stability and cell compatibility to regulate the proliferation of SMCs and ECs. The nanoparticles were expected to be used to prevent restenosis after surgery.

High-density lipoprotein (HDL) is a kind of natural targeting nanoparticle. Its main function is to transport cholesterol in the human body. In addition, it also has the function of maintaining vascular homeostasis, similar to the role of NO. To combine the functions of NO and HDL for biomimetic therapy of CVD, Rink et al. synthesized an S-nitrosylated (SNO) phospholipid (1,2-dipalmitoyl-sn-glycero-3-phosphonitrosothioethanol), which could be assembled with S-containing phospholipids, apolipoprotein A-I (the principal protein of HDL), and Au NPs as a template, to construct NO-delivering HDL-like particles (SNO HDL NPs) [109]. SNO HDL NPs can reduce ischemia/reperfusion injury in the mouse kidney transplantation model and decrease atherosclerotic plaque formation in the mouse atherosclerosis model.

**Figure 7:**
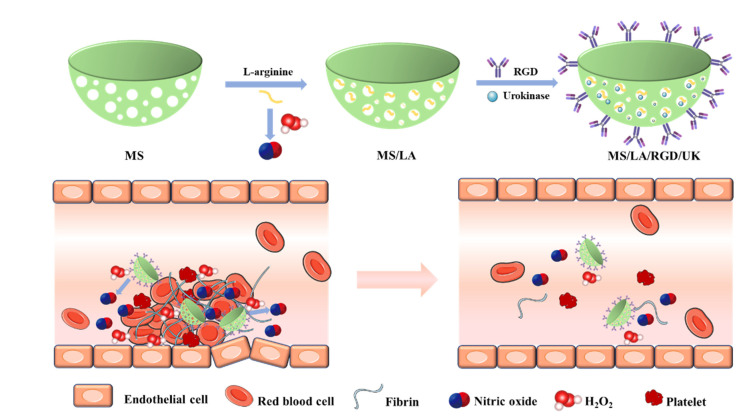
The preparation process of MS/LA/RGD/UK nanomotors and their application in the treatment of thrombosis. Reproduced with permission from [110], Journal of Colloid and Interface Science, 2022.

Because of the excessive blood flow rate, the drug utilization rate at the thrombus site is low in traditional therapy. To solve this problem, Tao et al. developed a biocompatible NO-driven silica nanomotor by loading L-arginine (LA) and thrombolytic drug urokinase (UK) in bowl-shaped mesoporous silica NPs (MS) and modifying the arginine–glycine–aspartic acid (RGD) polypeptide on the surface of NPs for targeting the thrombus surface (Figure 7) [110]. The loaded L-arginine can be oxidized by excessive ROS in the thrombus microenvironment to generate NO, thus decreasing oxidative stress in inflammatory endothelial cells by eliminating ROS. Meanwhile, the sustained release of NO not only promoted the movement and penetration ability of nanomotors to thrombosis sites but also improved the process of endothelialization. This therapeutic agent based on nanomotor technology is expected to support future research on thrombotic therapy.

### 3.5. Hydrogel

For tissue repair following a myocardial infarction, macrophage infiltration and the underlying inflammatory response are essential. The activated macrophages increase the expression and activity of iNOS, which generates NO when L-arginine is provided as substrate. Based on this background, Vong et al. synthesized a triblock copolymer (PArg-PEG-PArg) that incorporates L-arginine as a NO donor to treat myocardial infarction [111]. Under physiological conditions, the PArg-PEG-PArg copolymer can convert to hydrogel by its temperature-responsive property, facilitating the in situ release of NO, which can retain at the injected site for more than 10 days. The converted PArg–PEG–PArg hydrogel significantly reduced the infarction size and enhanced heart function in myocardial infarction model mice by improving angiogenesis and new blood vessel formation.

**Figure 8:**
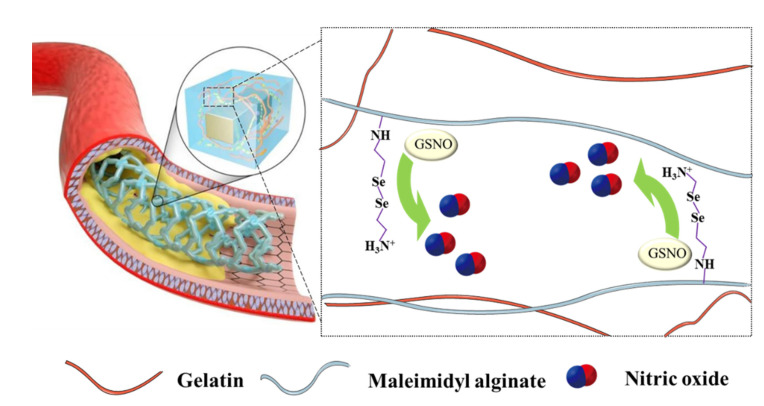
Schematic diagram of NOE hydrogel. Hybrid hydrogels made up of gelatin and maleimide-modified alginate, which are conjugated with SeCA to produce NO by catalyzing endogenous GSNO. The hydrogels could be used as coating for vascular stents. Reproduced with permission from [112], Nature Communications, 2021.

Besides adhering to pathological sites of tissues, hydrogels can be used as the coating and deposited on medical devices such as implants or stents. Chen et al. designed a nitric oxide-eluting (NOE) hydrogel coating for the surface modification of vascular stents [112]. The NOE hydrogel consisted of gelatin and alginate, which acted as backbones for conjugating with SeCA to produce NO by catalyzing endogenous NO donor GSNO (Figure 8). The hydrogel exhibited good mechanical toughness to withstand balloon expansion during angioplasty. More importantly, NO released from the hydrogel improved the adhesion of ECs and inhibited the excessive proliferation of VSMCs. The NOE hydrogel also modulated inflammatory responses and induced VSMCs relaxation, as revealed by transcriptome analysis. The NOE hydrogels were able to improve rapid endothelium regeneration, inhibit intimal hyperplasia, and regulate the inflammatory response in porcine coronary arteries, showing superior efficacy as compared to DESs.

NO-releasing biomaterials offer a considerable advantage over traditional approaches to clinical problems, such as short duration of action and poor targeting. However, these studies are still in the experimental or preclinical phase. The toxicity issue constrains the use of NO-releasing biomaterials in the clinic. By-product accumulation of biomaterials may exceed the tolerable limit, causing undesired toxicity. Therefore, it is important to take the formation and leaching of toxic by-products into consideration during the material design process.

## 4. Methods to Improve the Efficacy of NO-Based Therapy for CVD

Although NO-based therapy has achieved some success in the clinic or in preclinical animal experiments, novel strategies are still required in order to further improve efficacy. Combinatorial therapies exhibit synergistic efficacy and may overcome shortcomings, such as poor efficacy and side effects, found in single-agent treatments. Here, we introduce two methods, expected to augment the efficacy of NO-based therapy, including the combination of a NO-releasing platform with H_2_S-based therapy and stem cell therapy.

### 4.1. Synergistic Gas Therapy Based on NO and H_2_S

H_2_S, another kind of gasotransmitter, has similar characteristics to NO and performs biological functions in a variety of physiological systems [113]. Especially in recent years, it has been found that H_2_S can also be used in the treatment of CVD. For example, H_2_S can reduce the myocardial infarction area by activating Akt, PKC, and eNOS pathways, and decrease myocardial injury caused by myocardial ischemia-reperfusion by restoring mitochondrial function [114,115,116]. Kang et al. reported that exogenous H_2_S also can protect the heart through its anti-myocardial fibrotic activity [117]. In addition, H_2_S has effects on vasodilation, maintaining homeostasis in the cardiovascular system, and improving the sensitivity of vascular pressure reflex [118,119]. Besides the direct effect on the cardiovascular system, H_2_S can increase the NO production of ECs by activating eNOS [120]. In turn, NO can also exert this role in the generation of H_2_S [121]. At present, more and more evidence has shown that there are crosstalk effects between H_2_S and NO [122,123]. Especially, H_2_S can contribute to angiogenesis and vasorelaxation as an enhancer of vascular NO signaling [124]. Therefore, the synergistic effect of NO and H_2_S could be used to treat CVD.

**Figure 9:**
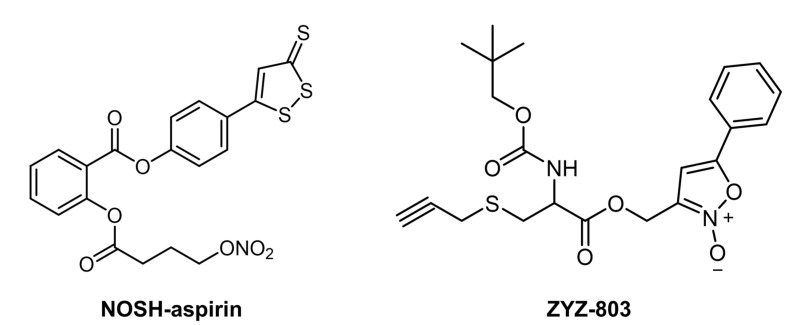
The chemical structures of NOSH-aspirin and ZYZ-803.

Recent studies have proven that H_2_S and NO donors can jointly act on CVD. Sun et al. observed that the reperfusion treatment with NaHS (H_2_S donor) can significantly reduce the systolic dysfunction and infarct area after ischemia. The combined use of NaHS and SNAP for reperfusion treatment exhibited an additive cardioprotection effect, which proved that the synergistic release of H_2_S and NO would bolster the treatment effects of CVD [114]. Additionally, researchers have developed multiple gas donors that can release these two gasotransmitters. Previous studies have reported that the backbone of nonsteroidal anti-inflammatory drugs (NSAID) was covalently attached with a NO-releasing and an H_2_S-donating moiety, resulting in a new class of anti-inflammatory pharmaceuticals, termed NOSH-NSAIDs, which can release both NO and H_2_S [125,126]. Similar to its parent compound, aspirin, NOSH-aspirin showed strong anti-inflammatory properties, anti-pyretic and analgesic effects, as well as antiplatelet activities (Figure 9) [125,127].

In recent years, ZYZ-803, a new dual NO-H_2_S donor, has been developed by combining the H_2_S-releasing part (S-propargyl-cystine, SPRC) and NO-releasing part (furoxan) (Figure 9) [128]. Zhu and coworkers observed that ZYZ-803 can slowly release H_2_S and NO under physiological conditions, and its cardiac protective effect is much higher than that of SPRC and/or furoxan [129]. Results of this study indicated that ZYZ-803 can regulate vascular tension and achieve effective, stable, and lasting vasodilation by increasing the concentration of vascular endothelial growth factor (VEGF) and cGMP and opening the KATP channel, proving that H_2_S and NO have a synergistic effect on regulating vascular tension. Zhu and coworkers also reported that ZYZ-803 had the ability to reduce LV remodeling and dysfunction in the development of heart failure and broaden the treatment of heart failure [130]. Cardioprotective effects of ZYZ-803 exceeded those of H_2_S and/or NO donor alone. In addition, ZYZ-803 also significantly improved angiogenesis by increasing the phosphorylation level of STAT3 (Tyr705) and CaMKII (Thr286) in HUVECs [131]. After treatment by ZYZ-803, significant increases in the blood perfusion and blood vessel density were observed in a hind limb ischemia mice model. This not only further revealed the beneficial role of ZYZ-803 in STAT3/CaMKII-related CVD but also had implications for the treatment of lower extremity vascular ischemic diseases. Although the synergistic gas therapy based on H_2_S and NO has been successful to a certain extent, the underlying therapeutic mechanism is not fully elucidated. Much work needs to be carried out to investigate the cross-talk between H_2_S and NO and clarify the specific targets of gaseous signaling molecules in CVD. It is believed that synergistic gas therapy has a very broad development prospect in treatments for CVD.

### 4.2. Synergistic Therapy Based on Stem Cells and NO

Combining stem cells and NO-releasing biomaterials is an effective and holistic method to improve tissue regeneration and overcomes the limitations of the individual components. Both stem cells and NO can actually interact, which would in turn increase their activities. Numerous studies have demonstrated that mesenchymal stem cells (MSCs) can improve local NO levels and thus restore endothelial function [132]. One of the studies confirmed that MSCs derived from skin were capable of enhancing NO production by secreting VEGF, thereby resulting in increased vasodilation [133]. Furthermore, MSCs can paracrine factors that are able to increase eNOS expression in the endothelium as well as to decrease iNOS immunoreactivity through regulation of macrophage polarization in inflammatory lipid cores, thus decreasing the risk of plaque rupture [134,135,136]. In addition, ECs can be protected by stem cell-derived exosomes by improving NO production [135,137,138]. In light of the function of stem cells in regulating endothelial function by upregulating the expression of eNOS and inhibiting inflammation by reducing iNOS expression, it is conceivable to regard stem cells as a powerful and effective method of treating CVD.

Researchers have reported that NO released from biomaterials can affect the function of stem cells, including changes in paracrine secretion patterns, release of growth factors, and generation of exosomes [139]. Du et al. studied the effect of a NO-releasing polymer on the exosome secretion of human placenta-derived MSCs (hP-MSCs) and found that NO can significantly increase the concentration of VEGF in the exosomes, thus enhancing the angiogenesis of HUVECs [140]. The discovery of this mechanism is of great significance for the treatment of myocardial infarction. In addition, the combination of eNOS overexpression and stem cell transplantation can be used to treat myocardial infarction [141,142,143]. Chen et al. found that the myocardial parameters were improved, the infarct area was reduced, and the number of new capillaries was increased in rats with myocardial infarction treated by eNOS highly expressed MSCs, which was superior to the treatment with eNOS gene or stem cells alone, showing the advantage of “one plus one over two” [144]. Therefore, they concluded that ectopic high expression of eNOS in vivo can enhance the ability of MSCs to treat ischemic heart injury after coronary artery occlusion.

Adipose-derived stem cells (ADSCs) have the important ability of MSCs, namely, differentiation into cardiomyocytes, ECs, and VSMCs [145]. Additionally, ADSCs possess stronger abilities of proliferation, differentiation, and VEGF secretion than bone marrow mesenchymal stromal cells [146,147]. However, the treatment by ADSCs, like most stem cell transplantations, still has the problems of poor donor cell implantation and low survival rate [148]. To solve these problems, hydrogels are absolutely the best choice for synergistic therapy of NO and stem cells, due to their ability to mimic the native extracellular matrix (ECM), which can support cell survival and coordinate stem cell repair of functional tissues [149]. Zhao and colleagues developed a naphthalene-peptide hydrogel containing β-galactose-caged NONOate (NapFF-NO), which release*s* NO only in the presence of β-galactosidase [150]. NapFF-NO was co-transplanted with ADSCs into myocardial infarction mice models and β-galactosidase was injected into the tail vein at the same time. The treatment improved cell survival within the engraftment site and fostered a microenvironment enriched with VEGF and stromal cell-derived factor-1α (SDF-1α) to facilitate ECs migration and improve heart function. Besides polypeptide, Zhao and colleagues also used chitosan as a backbone to graft galactose-caged NO donors, and then the injectable hydrogel (CS-NO) was obtained [151]. CS-NO enhanced hP-MSC angiogenic and ischemic potential through controlled NO release [140]. Moreover, NO released from CS-NO can also induce embryonic stem cells (ESCs) to differentiate into ECs in the case of not adding growth factor, thereby providing a feasible method for EC-based therapies in vascular repair [152].

**Figure 10:**
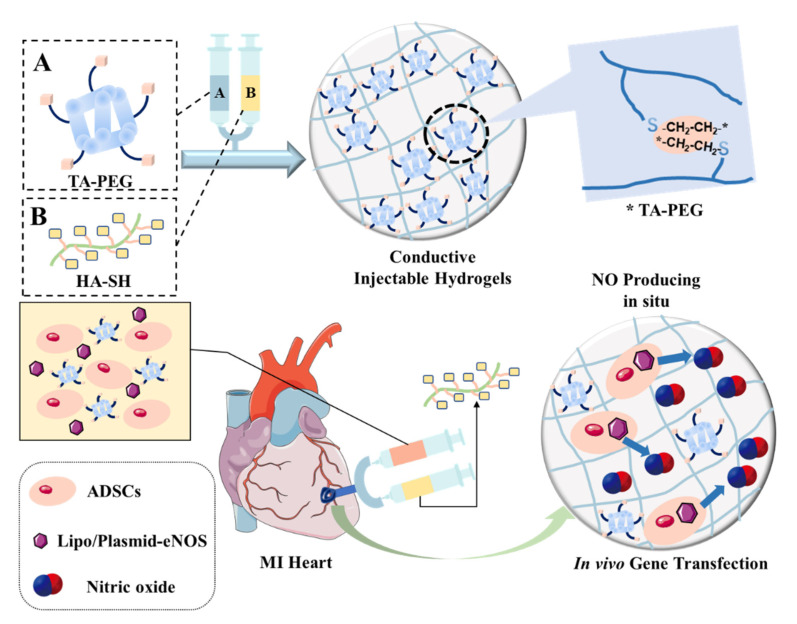
Schematic diagram of the conductive injectable hydrogel, which is employed to load plasmid DNA-eNOs nanoparticles and ADSCs to treat myocardial infarction. “A” represents TA-PEG, and “B” represents HA-SH. Reproduced with permission from [153], Biomaterials, 2018.

Although this synergistic therapy has achieved a remarkable performance, NO-releasing hydrogels have the disadvantage of a limited supply of NO, which is unfavorable to the long-term treatment. Wang et al. designed a conductive hydrogel composed of tetraaniline-polyethylene glycol diacrylate (TA-PEG) and thiolated hyaluronic acid (HA-SH), which could form a hydrogel in situ (Figure 10) [153]. The hydrogel was used to load ADSCs and nanocomplexes containing plasmid DNA encoding eNOS, combining stem cell and gene therapy to overcome the limited payload of NO in the hydrogel. These hydrogels were applied in the treatment of myocardial infarction, showing a sustained release of NO to the myocardium, thus maintaining a microenvironment conducive to regeneration. Consequently, the infarct size and fibrosis were reduced, and the heart function was improved significantly. The synergistic therapy with stem cells and a NO-releasing platform has great potential for use in the treatment of CVD because it can not only maintain the survival of stem cells injected into the body but also stabilize the production of NO, which has broad prospects. However, the synergistic therapy based on stem cells and NO still faces a bottleneck in the development of NO-releasing biomaterials, which should be suitable for the co-delivery of stem cells and extend the period of NO release in an on-demand manner during treatment. It is expected that the use of NO donors in stem cell-mediated therapies will increase as new NO donors are discovered and synthesized.

## 5. Conclusions and Outlook

Because CVD is a kind of disease with a high mortality rate in the world, many studies have been dedicated to exploring efficient drugs or therapeutic devices for CVD. As a representative gasotransmitter in the cardiovascular system, NO has shown beneficial effects for the treatment of CVD, and thus attracted many researchers to develop different NO delivery strategies based on various molecular structures and chemical materials that enable controllable NO release. It is clear that some progress has been made in recent years. However, there are still many problems to be addressed. NO concentration plays a key role in determining therapeutic effects, and inappropriate concentrations may lead to further deterioration of the disease. In order to treat CVD by promoting angiogenesis and inhibiting thrombosis, a mild and long-term release of NO is desirable. To give an example, catalysts, such as Cu-MOF, can produce mild levels of NO for long-term use in CVD therapies. Moreover, NO prodrugs that respond to enzymes can tune the rate of NO release and increase specificity and potency at low NO doses. Additionally, future studies should focus on developing microenvironment-responsive NO donors and biomaterials, which are capable of sensing pathological microenvironments of CVD, and decomposing to produce NO in an on-demand and controlled manner. Another concern is that the substrate materials that cover stents, catheters, vascular grafts, and nanoparticles should minimize toxicity.

To achieve better treatment of CVD, it is important that NO is delivered to the patient’s target tissues with the expected dose, location, and time. By using computational modeling, researchers can evaluate the spatial distribution of NO at pathological locations, thus enabling the NO-releasing platform to be targeted to the regions that have lower local NO concentrations than those in healthy states. In addition, biomaterial-based delivery systems equipped with both diagnostic and therapeutic functions can be used to monitor NO release as well as visualize the evolution of diseases, which can serve for checking the efficacy of the treatment and provide valuable information for doctors and patients to make optimal treatment decisions.

Although NO has long been established as an independent signaling molecule, recent studies have shown that H_2_S enhances NO in the cardiovascular system. Gaseous signaling molecules released from dual NO-H_2_S donors, such as ZYZ-803, can be far more effective at treating CVD compared to individual administration of drugs with single gas signaling molecules. At present, a number of donors have been identified, but it remains unclear what concentration of gaseous signaling molecules is present in various samples, making the process more difficult. Thus, the need for an accurate and appropriate method is urgent. Furthermore, it is also extremely important to determine the specific targets of gaseous signaling molecules in CVD and to evaluate these features in clinical applications. It is anticipated that with the progress of scientific research and continuous efforts of researchers, gaseous signaling drugs will soon enter clinical research.

Recent studies have revealed how NO affects the behavior of stem cells and regulates the microenvironment. This, in turn, enhances the therapeutic effect and action region of NO by affecting nearby resident stromal and immune cells to create a highly regenerative tissue state. NO-releasing biomaterials should be suitable for delivering stem cells, preserving cell survival, and extending NO release during treatment with on-demand mechanisms. With the continuous research and development of NO-releasing platforms, we believe that the synergistic therapy based on NO and stem cells will make great breakthroughs in the treatment of CVD.

In summary, NO-related gas molecular therapy and synergistic therapy are constantly evolving, and major strides have been made to treat CVD. NO donors and NO-related drugs have been commonly administered for the treatment of CVD in the clinic. While NO-releasing biomaterials are still in the experimental or preclinical phase, many studies have yielded encouraging results. In addition, the combination of a NO-releasing platform with H_2_S-based therapy and stem cell therapy has also aroused great interest and shown promising therapeutic effects, but much work is still in the early exploration and experimental research stage. It is hoped that the continuous development of NO-releasing platforms will advance the treatment strategies of CVD and can be available routinely in clinical applications.

## Figures and Tables

**Table 1:** Summary of the NO donors or NO-related drugs for the treatments of CVD.

Name	Essence	Feature	Application	Refs.
Sodium nitroprusside	NO donor	A direct NO donor independent of the endothelium.	Rapid blood pressure reduction	[28]
Clofibrate	PPARα agonist (indirectly increases NO prodution)	Increases eNOS protein expression and enzyme activity in the left ventricle.	Treatment of hypertension	[29]
Nitroglycerin	NO donor	Suppresses ST segment elevation and reduce infarct size by promoting the development of collateral coronary circulation	Controls angina pectoris and treats myocardial infarction	[30,31]
Amyl nitrite	NO donor	Release NO after the formation of S-nitrosothiol intermediate by interaction with sulfhydryl groups	Lowers blood pressure and treats angina pectoris	[32,33]
Molsidomine	NO donor	Regulate NO-cGMP signaling via multiple pathways in vivo	Maintains atherosclerotic plaque stability and prevents myocardial infarction and congestive heart failure	[34,35]
Rosiglitazone	PPARγ agonist (indirectly increases NO by activating the eNOS)	Upregulates nuclear factor erythroid-2-related factor 2 (Nrf2) in the positive feedback loop to maintain transcription factor expression	Treatment of spontaneous hypertension in youth	[36]
Nebivolol	Third-generation β-receptor blocker (indirectly increases NO prodution)	The β-adrenergic antagonist that causes vasorelaxation primarily by activating eNOS	Treatment of LV dysfunction of myocardial infarction; antioxidant properties, preventing NOS uncoupling; improving LV function in chronic heart failure	[37,38,39,40,41,42]
Fenofibrate	PPARα agonist (indirectly increases NO prodution)	Increases endothelial eNOS expression; prevents endothelial dysfunction; reconnects eNOS	Protecting atherosclerosis and endothelial dysfunction, and finally preventing myocardial infarction	[43,44,45]
LA419	NO donor	The effects of anti-ischemia, anti-thrombosis and anti-atherosclerosis	Treatment of myocardial infarction; the prevention of the progression of inadaptable cardiac hypertrophy	[28,46]
L-arginine	A precursor of NO production	Reversing several markers in LV hypertrophy and reducing blood pressure and interacting with renin–angiotension–aldosterone system and sympathetic nervous system and other systems	Prevents the progression of LV hypertrophy, anti-hypertrophic effect	[47,48,49]
BRL37344	β_3_-ARs agonists (indirectly increases NO prodution)	Increases nNOS protein expression and NO production, inhibits superoxide anion generation	Improves LV systolic and diastolic dysfunction, used in the treatment of cardiac hypertrophy	[37]
Carbachol	M-cholinergic agonist (indirectly increases NO prodution)	Mediates the production of NO from nNOS	Prevents heart failure	[50]

## Data Availability

Not applicable.
